# Supplementary material for: Association between oral microbiome and seven types of cancers in East Asian population: a two-sample Mendelian randomization analysis
Source: Front Mol Biosci. 2023 Nov 21;10:1327893. doi: 10.3389/fmolb.2023.1327893 (PMC10702768; doi:10.3389/fmolb.2023.1327893)
Supplement: Supplementary file 1 [file DataSheet1.ZIP › Supplementary Materials/MR plots for tongue/tongue═╝/Ovarian cancer/pheno.3_to_ovarian cancer_forest.pdf]

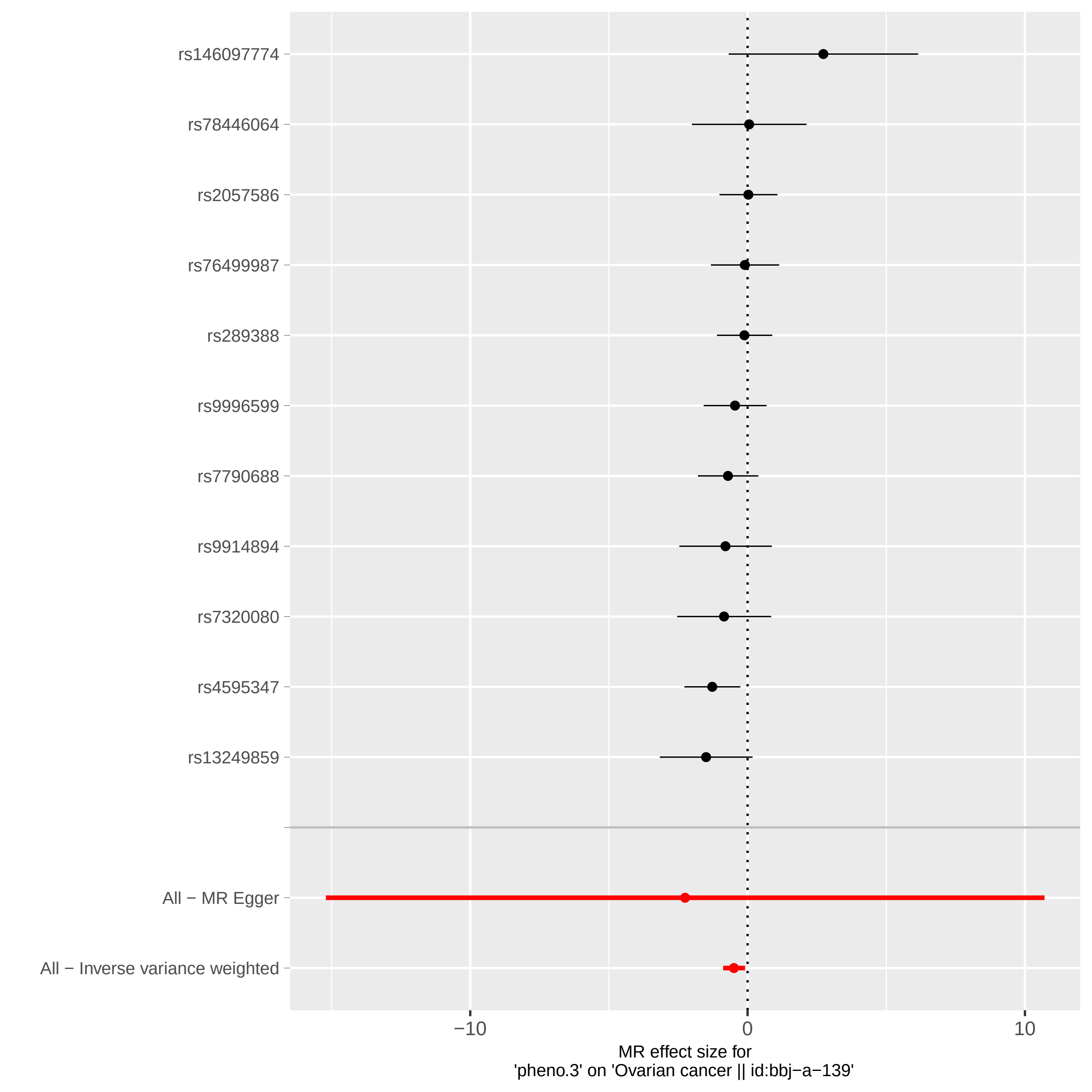
rs146097774
rs78446064
rs2057586
rs76499987
rs289388
rs9996599
rs7790688
rs9914894
rs7320080
rs4595347
rs13249859
All − MR Egger
All − Inverse variance weighted
−10
0
10
MR effect size for
'pheno.3' on 'Ovarian cancer || id:bbj−a−139'
